# Supplementary material for: Zinc mediates control of nitrogen fixation via transcription factor filamentation
Source: Nature. 2024 Jun 26;631(8019):164–9. doi: 10.1038/s41586-024-07607-6 (PMC11222152; doi:10.1038/s41586-024-07607-6)
Supplement: Supplementary file 2 — Reporting Summary [file 41586_2024_7607_MOESM2_ESM.pdf]

nature portfolio

Corresponding author(s): Dugald Reid, Kasper Andersen, Jieshun Lin

Last updated by author(s): May 17, 2024

Reporting Summary

Nature Portfolio wishes to improve the reproducibility of the work that we publish. This form provides structure for consistency and transparency in reporting. For further information on Nature Portfolio policies, see our Editorial Policies and the Editorial Policy Checklist.

Statistics

For all statistical analyses, confirm that the following items are present in the figure legend, table legend, main text, or Methods section.

n/a | Confirmed

- [ ] | [x] The exact sample size (*n*) for each experimental group/condition, given as a discrete number and unit of measurement
- [ ] | [x] A statement on whether measurements were taken from distinct samples or whether the same sample was measured repeatedly
- [ ] | [x] The statistical test(s) used AND whether they are one- or two-sided
Only common tests should be described solely by name; describe more complex techniques in the Methods section.
- [ ] | [x] A description of all covariates tested
- [ ] | [x] A description of any assumptions or corrections, such as tests of normality and adjustment for multiple comparisons
- [ ] | [x] A full description of the statistical parameters including central tendency (e.g. means) or other basic estimates (e.g. regression coefficient) AND variation (e.g. standard deviation) or associated estimates of uncertainty (e.g. confidence intervals)
- [ ] | [x] For null hypothesis testing, the test statistic (e.g. *F*, *t*, *r*) with confidence intervals, effect sizes, degrees of freedom and *P* value noted
Give P values as exact values whenever suitable.
- [x] | [ ] For Bayesian analysis, information on the choice of priors and Markov chain Monte Carlo settings
- [x] | [ ] For hierarchical and complex designs, identification of the appropriate level for tests and full reporting of outcomes
- [x] | [ ] Estimates of effect sizes (e.g. Cohen's *d*, Pearson's *r*), indicating how they were calculated

Our web collection on statistics for biologists contains articles on many of the points above.

Software and code

Policy information about availability of computer code

Data collection	No software was used for data collection
Data analysis	FUN protein sequences were identified by BLAST 2.15.0 and SHOOT 1.1.0 and aligned with MAFFT 7.490 and a tree constructed using FastTree 2.1.11. The tree was visualised using iTOL 6.7.3. RNAseq analysis was performed by mapping reads to the reference transcriptome using Salmon 1.10.1 and quantification performed using DEseq2 1.40.2. Elemental distribution was analysed using PyMca 5.9.2

For manuscripts utilizing custom algorithms or software that are central to the research but not yet described in published literature, software must be made available to editors and reviewers. We strongly encourage code deposition in a community repository (e.g. GitHub). See the Nature Portfolio guidelines for submitting code & software for further information.

Data

Policy information about availability of data

All manuscripts must include a data availability statement. This statement should provide the following information, where applicable:

- Accession codes, unique identifiers, or web links for publicly available datasets
- A description of any restrictions on data availability
- For clinical datasets or third party data, please ensure that the statement adheres to our policy

A reporting summary for this article is available as Supplementary Information file. The main data supporting the findings of this study are available within the article, its Extended Data Figures and supplementary information files. RNAseq raw data has been submitted to NCBI under accession PRJNA985805 and processed

data with differential expression statistics is available as supplementary file 1. Source data for each figure are provided as supplementary information files. Full versions of EMSA blots are included in the source data file.

Research involving human participants, their data, or biological material

Policy information about studies with human participants or human data. See also policy information about sex, gender (identity/presentation), and sexual orientation and race, ethnicity and racism.

Reporting on sex and gender	not applicable
Reporting on race, ethnicity, or other socially relevant groupings	not applicable
Population characteristics	not applicable
Recruitment	not applicable
Ethics oversight	not applicable

Note that full information on the approval of the study protocol must also be provided in the manuscript.

Field-specific reporting

Please select the one below that is the best fit for your research. If you are not sure, read the appropriate sections before making your selection.

☒ Life sciences ☐ Behavioural & social sciences ☐ Ecological, evolutionary & environmental sciences

For a reference copy of the document with all sections, see nature.com/documents/nr-reporting-summary-flat.pdf

Life sciences study design

All studies must disclose on these points even when the disclosure is negative.

Sample size	Sample size was not predetermined. Sample sizes were in line with norms of the field and determined by previous experience i n performing experiments of the same technical nature. For each experiment this depended on previous experience with the biological and technical variation inherent in each technique.
Data exclusions	The linear data points in the Guinier plots (closed circles) were used in the fit. Non-linear data points in the Guinier plots (open circles) were omitted. The criteria for excluding the non-linier data points were pre-established. No other data was omitted in this study.
Replication	Independent biological replicates were used in all experiments and indicated as n in figure legends, with all replications successful. Technical replication was performed in qPCR which was averaged before comparing biological replicates
Randomization	All research material was homozygous, and therefore individual organisms were randomly distributed to control and treatment groups
Blinding	Experimenters were not blind to sample collection, but tubes were assigned numbers which were reconciled with sample names once molecular analysis was completed

Reporting for specific materials, systems and methods

We require information from authors about some types of materials, experimental systems and methods used in many studies. Here, indicate whether each material, system or method listed is relevant to your study. If you are not sure if a list item applies to your research, read the appropriate section before selecting a response.

Materials & experimental systems

n/a	Involved in the study	
☒	☐	Antibodies
☒	☐	Eukaryotic cell lines
☒	☐	Palaeontology and archaeology
☒	☐	Animals and other organisms
☒	☐	Clinical data
☒	☐	Dual use research of concern
☐	☒	Plants

Methods

n/a	Involved in the study	
☒	☐	ChIP-seq
☒	☐	Flow cytometry
☒	☐	MRI-based neuroimaging

Plants

Seed stocks	All Lotus seeds were obtained from lotus base available at lotus.au.dk Line numbers are available in extended data file 2
Novel plant genotypes	The fun mutant was screened as described in methods from a population of Lotus individuals carrying randomly inserted LORE1 insertions. Insertion sites were characterised by insertion sequencing and PCR validation as described in methods
Authentication	Mutants were verified by PCR genotyping as described in the methods section using primers listed in extended data figure 2
